# Evidence Map of Prevention and Treatment Interventions for Depression in Young People

**DOI:** 10.1155/2012/820735

**Published:** 2012-03-15

**Authors:** Patrick Callahan, Ping Liu, Rosemary Purcell, Alexandra G. Parker, Sarah E. Hetrick

**Affiliations:** ^1^Orygen Youth Health Research Centre, Centre for Youth Mental Health, The University of Melbourne, Locked Bag 10, Parkville, Victoria 3052, Australia; ^2^Headspace Centre of Excellence, The National Youth Mental Health Foundation, P.O. Box 473, North Melbourne, Victoria 3051, Australia

## Abstract

*Introduction.* Depression in adolescents and young people is associated with reduced social, occupational, and interpersonal functioning, increases in suicide and self-harm behaviours, and problematic substance use. Age-appropriate, evidence-based treatments are required to provide optimal care. *Methods.* “Evidence mapping” methodology was used to quantify the nature and distribution of the extant high-quality research into the prevention and treatment of depression in young people across psychological, medical, and other treatment domains. *Results.* Prevention research is dominated by cognitive-behavioral- (CBT-) based interventions. Treatment studies predominantly consist of CBT and SSRI medication trials, with few trials of other psychological interventions or complementary/alternative treatments. Quality studies on relapse prevention and treatment for persistent depression are distinctly lacking. *Conclusions.* This map demonstrates opportunities for future research to address the numerous evidence gaps for interventions to prevent or treat depression in young people, which are of interest to clinical researchers, policy makers, and funding bodies.

## 1. Introduction

Depression among adolescents and young people is a pressing public health issue. A meta-analysis of prevalence rates shows 2.8% of children (<13 years) and 5.7% of adolescents (13–18 years) experience depression over a 12-month period [1]. Depression has serious adverse effects on social, academic, and family functioning [2, 3]. Early onset depression is also associated with higher rates of completed suicide and suicide attempts compared to individuals with no psychiatric illness [4, 5].

High prevalence and the associated disability of depressive disorders make effective, evidence-based prevention and early interventions a priority. While the status of depressive disorders as a public health priority due to the associated burden of disease [6] has ensured a large amount of effort has gone into prevention research, an equally important, but neglected focus has been on early intervention for those with emerging or first episode depression.

The age of onset for most disorders, including depressive and anxiety disorders, falls in a narrow time band, predominantly encompassing youth from the early teens to the mid 20's [7]. Early detection and intervention is critical to preventing a relapsing or chronic course, which is associated with the development of other psychiatric and alcohol and substance use disorders [8, 9], impairments in occupational and social functioning [3, 10–13], and the risk of suicide [5, 14–16]. Harrington and Clark [17] demonstrated that if all cases of depression in those aged 13 were treated successfully then the risk of depressive disorder at age 16 years would be reduced by approximately 10%.

The idea of intervening early to assertively and effectively treat depression and prevent the development of a relapsing or chronic course is consistent with the clinical staging model for psychiatry [18] that proposes that those in the early stages of an illness respond better to treatment and thus require more benign interventions whereas those with a more developed disease process will require more complex and perhaps invasive interventions [18]. Clinical staging is a more refined form of diagnosis, placing emphasis on where a person lies along the continuum of the course of illness [18–22]. While yet to be fully realized, it provides a framework for early intervention guiding the use of appropriate stage-specific evidence based treatments, including preventatively orientated treatments [18, 21, 22]. In the case of major depression, research demonstrates that intervening during these earlier stages is clinically warranted given the considerable impairment associated with “subthreshold” depression, which is equally, if not more, prevalent than the diagnosable disorder [11, 23].

This framework for early intervention relies on the effective implementation of evidence-based practice. However, evidence-based interventions are far from universally delivered, with research indicating, for example, that antidepressant medications with minimal empirical support are prescribed to young people in high numbers [24, 25]. Having access to, and being familiar with, the best available evidence is a critical step in improving the uptake of evidence-based practice.

One approach to summarising the large volume of existing evidence is to undertake literature reviews. This process has resulted in an array of different terminologies and methodologies to describe approaches that share similar characteristics, namely, collecting, evaluating, and presenting the available research evidence [26]. Systematic reviews are the most methodologically sound, as they use explicit methods of searching, appraising, and analyzing data to address a focused clinical question [27]. Although methodologically rigorous and providing useful summaries of the existing published and unpublished data, they do not allow readers a meaningful overview of all treatments across a population with similar presentations, which has potential relevance to a new and emerging field such as youth mental health.

Evidence mapping is a process of reviewing the literature in a manner that enables the breadth of the research activity in a particular field to be explored. While systematic reviews can address specific clinical questions (e.g., is treatment X effective for condition Y?), evidence mapping allows a concise summary of the extent and distribution of evidence in a broad field of interest (e.g., what interventions are available for condition Y?). Evidence maps are based on an explicit research question relating to the field of enquiry, which may vary in depth, but should be informed by end users. The research question then drives the search for, and collection of, appropriate studies utilising explicit and reproducible methods at each stage [26, 28, 29]. This includes clear definition of components of the research question, development of a thorough and reproducible search strategy, development of explicit inclusion and exclusion criteria, and transparent decisions about the level of information to be obtained from each study. The end-user may be researchers or research funding bodies who can identify gaps in the evidence, which in turn will create opportunities for new research and policy makers, who will use the evidence map to inform policy decisions, or clinicians who can access information about interventions.

This paper presents the results of an evidence map we conducted on depression in young people. The extent, range, and nature of high-quality clinical research interventions for depression in young people is summarised. This process of taking stock of the evidence is an essential first step in obtaining an overview of the breadth of research activities before further exploring the effectiveness of interventions.

## 2. Materials and Methods

The depression evidence map was produced as a part of a larger evidence mapping project undertaken by the Centre of Excellence in Youth Mental Health (part of *headspace;* the Australian National Youth Mental Health Foundation; http://www.headspace.org.au/). The centre is responsible for generating and disseminating evidence regarding interventions for adult-type mental health disorders that emerge during adolescence and young adulthood. A detailed description of the methodology for conducting these evidence maps has been published elsewhere [28]; however, the methods specific to the depression map are provided below.

**Step 1. Create a Map Based on Broad Clinical Questions Relating to the Field of Enquiry**

After consulting with expert research mental health clinicians (psychiatrists and psychologists) in depression prevention and intervention at Orygen Youth Health Research Centre and *headspace,* the questions and scope of the evidence map were defined. This process revealed two areas of focus for the map, namely;

- What good-quality evidence exists regarding prevention of and interventions for emerging or established depressive disorders among young people?
- What areas are, and are not, well researched?

**Step 2. Define Key Variables, Specifying Characteristics to Be Mapped and Develop Inclusion and Exclusion Criteria**

Based on these key questions, the characteristics of studies to be included in the map were defined, encompassing the population, types of intervention, and phases of depressive illness [28]. Intervention studies that had as their sample adolescents and young adults aged 12–25 years old with a depressive disorder, classified by the DSM-IV or ICD-10 as the primary diagnosis, were included regardless of the methods of assessment of depressive disorder. Studies where participants were recruited on the basis of elevated risk factors or subthreshold/subsyndromal presentations of depression were also included and again the methods of assessment varied across trials and were included regardless. In the context of appropriately mapping the prevention and early intervention research, prevention trials were including if participants were aged 6 years and older. Trials with both adult and adolescent participants were included if the mean age of participants of any intervention group was at 25 years of age or under. We excluded studies where participants were recruited on the basis of physiological or medical conditions (e.g., depression in the context of experiencing cancer or dementia). Interventions were defined as “anything delivered for the purpose of relieving symptomatology or improving functioning of the target disorder” [28].

Prevention trials were categorized into universal, selective, and indicated prevention. Universal interventions are those delivered to a designated population regardless of their risk; selected prevention interventions are those designed to be delivered to members of the population with a risk factor for a given disorder; indicated interventions are those delivered to populations with signs or symptoms of that disorder [30]. We combined selective and indicated prevention trials into one category in the evidence map that indicated populations “at risk.” The inclusion criteria for relapse prevention studies required that the study specify that the intervention was designed to prevent relapse or maintain improvements in patients who had previously responded to treatment. Subsequently, studies assessing an acute phase intervention with a long-term phase were categorized as a treatment study. In the depression literature, the definition of treatment resistance (or more appropriately, persistent depression) is an area of debate and an array of systems exist to categorize different levels of nonresponse to treatment [31]. As such, it was decided to include treatment-resistant studies where the authors labeled them as such. We have labeled these studies “persistent depression interventions” as “treatment resistance” is arguably a pejorative term that implies fault on the client's behalf for being “resistant” to treatment.

We sought to present “good-quality evidence,” and as such we only utilized evidence from RCTs, pseudorandomised controlled trials, clinical controlled trials, systematic reviews, and meta-analyses, since these are commonly considered the most robust study designs for examining the effectiveness of interventions [32]. Definitions of review types are not consistent and many different terms are used, at times interchangeably [26], therefore we included those reviews where a systematic search strategy was used. Included studies were published in English from 1980 to May 2009.

**Step 3. Searching the Literature**

Search strategies for MEDLINE, PSYCHINFO, EMBASE, and The Cochrane Central Register of Controlled Trials (CENTRAL) were devised using subject headings such as “depression,” “randomized controlled trial,” and “review” appropriate for each database. Additional free text words identified by experts were also included. The search was broad and inclusive as the depression literature lacks clear early intervention terms such as “ultra high risk” and “first episode” used in the early psychosis field [33].

A filter to identify relevant publication types based on the search strategy devised by Glanville et al. was also incorporated [34]. The overall structure of searches targeted and combined depression terms, study methodologies, stage of illness terms, and were limited by year and English language (a full search strategy is available upon request to the corresponding author). Search strategies were revised after a random sample of 100 citations retrieved for the search was examined, as well as cross-checking the retrieval for 20 articles known to meet the inclusion criteria.

**Step 4. Screening and Positioning the Relevant Evidence within the Map (e.g., Charting)**

The titles and abstracts of all potentially relevant papers identified by the searches of all databases were collated. All authors independently screened 100 references randomly selected from the search results as a pilot trial to examine the consistency of applying the inclusion and exclusion criteria. A satisfactory interrater reliability in excess of 0.90 was achieved for the pilot screening. All authors were involved in the screening of retrieved citations. Where a title or abstract reported a trial that appeared to be eligible for inclusion, the full article was obtained. All references retained after the initial screening were then assessed against the inclusion and exclusion criteria (PC) based on their full texts. Trials with no information on the age range or mean age of patients were excluded. Reviews summarising evidence of treatments for both adults and adolescents were included if there was specific content devoted to the adolescent and young adult population.

References that met the inclusion criteria were then coded according to the type of intervention, stage of depressive illness, and study types. Intervention types were generally classified into psychological, biological, integrated, complementary/alternative interventions and service/delivery improvement. The stage of illness was coded as universal prevention, selected/indicated prevention, disorder established, and relapse prevention. Publication types were coded as RCT/CCT (referred to as “trials” throughout results) and systematic review (referred to as “reviews” throughout results). Studies that evaluated the effectiveness of more than one type of intervention were coded for each intervention, thus the addition of the total number of studies from each coded section is greater than the total number of included studies.

The primary reference for each study was established with secondary publications indicated as such. This process prevented counting one study multiple times and misrepresenting the number of studies in a particular area. For example, the Treatment of Adolescents with Depression Study [35] has produced dozens of publications; however, it is a single trial with 439 participants.

The current study goes beyond the scope of the overall evidence mapping in that where a systematic review was available and included in the map, a short qualitative description of the main conclusions has been provided.

## 3. Results

### 3.1. Included Trials

**Figure 1:**
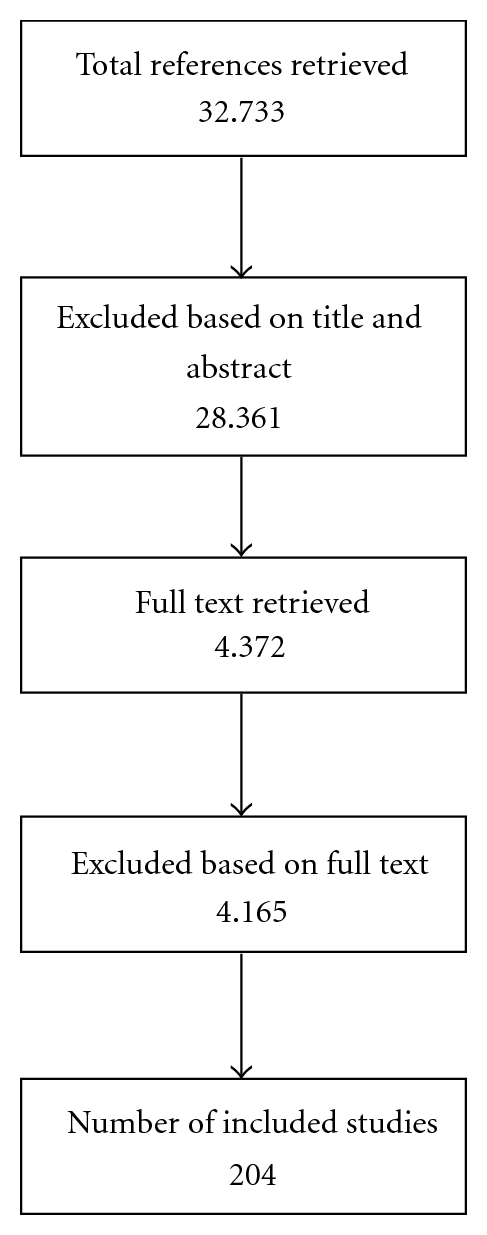
Distribution of included universal preventive studies.

Our search strategies identified 32,733 references, of which 4,372 potentially relevant references were retained based on the title and abstract. Full texts of these references were retrieved. Based on the information provided by the full text of the retrieved publications, 204 publications were included in the final map. These represent a total of 162 trials, as well as 41 systematic reviews and meta-analyses (see Figure 1). A list of citations for all RCTs and systematic reviews included in the map are available on request or the included studies can be found on our searchable database (http://www.headspace.org.au/what-works/evidence-maps).

### 3.2. Universal Prevention Studies

**Figure 2:**
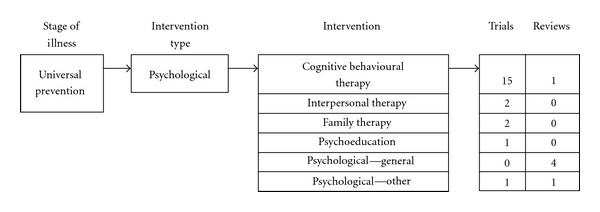
Flowchart for included studies.

A total of 23 universal prevention studies were identified, consisting of 18 RCTs and 5 systematic reviews (Figure 2). Of the 18 trials, 15 focused on cognitive behavioural therapy (CBT), many originating from the University of Pennsylvania (e.g., the Penn Prevention Programme and the Penn Resiliency Programme [36, 37]). The remaining trials utilized interpersonal (*n* = 2), family (*n* = 2), psychoeducation (*n* = 1), and leadership (*n* = 1) programs.

There were no universal prevention trials utilising complementary or alternative interventions. However, one Cochrane systematic review was included that explored the benefits of exercise as a preventative intervention.

Reviews of universal interventions are common and provide useful summaries of existing trials. The map includes four general reviews of various types of universal interventions, most commonly delivered in group format [38–41]. These reviews are generally cautious in their support of universal prevention programs for children and adolescents because such interventions were not consistently shown to be effective in a recent review (e.g., [41]). It has been suggested that this may be due to the low effect sizes typically demonstrated in universal prevention studies [41]. The reviews highlight the need for more research given some promising results (e.g., [42] in [41]) and in combination with the potential benefits of universal prevention programs that are less stigmatising than selected prevention interventions and potentially cost-effective ways to reduce depression in the community [41].

### 3.3. Selective and Indicated Prevention Studies

**Figure 3:**
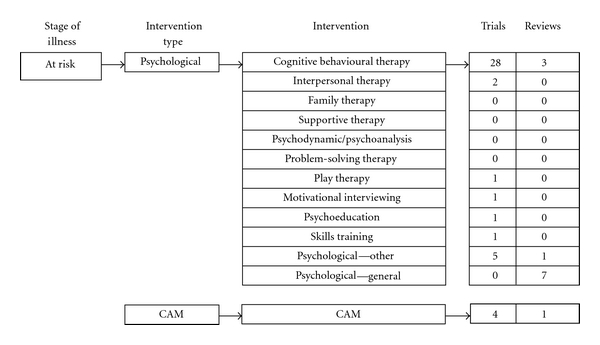
Distribution of included indicated and selective preventive studies.

In total, 54 studies were identified, consisting of 43 trials and 11 systematic reviews (Figure 3). The majority of research again focused on CBT-based interventions (*n* = 28). There were 2 studies of interpersonal psychotherapy (IPT), although only one involved IPT as the primary intervention. There was one study each of exercise, psychoeducation, play therapy, and skills training, and 4 complementary and alternative interventions (*n* = 4).

Seven reviews were included that assessed all types of interventions for those at risk of depression. Generally, targeted interventions, which were again mostly delivered in a group format, were found to be effective in preventing depression immediately after the delivery of interventions, although robust long-term effects are still to be shown [43–45]. CBT programs in particular have been consistently found to be effective in preventing depression in at-risk young people [41, 44, 45] with one review [41] highlighting the effectiveness of a CBT based program called “Coping with Depression,” even at long term followup [46, 47].

### 3.4. Interventions for Diagnosed Depression (Established Disorder)

**Figure 4:**
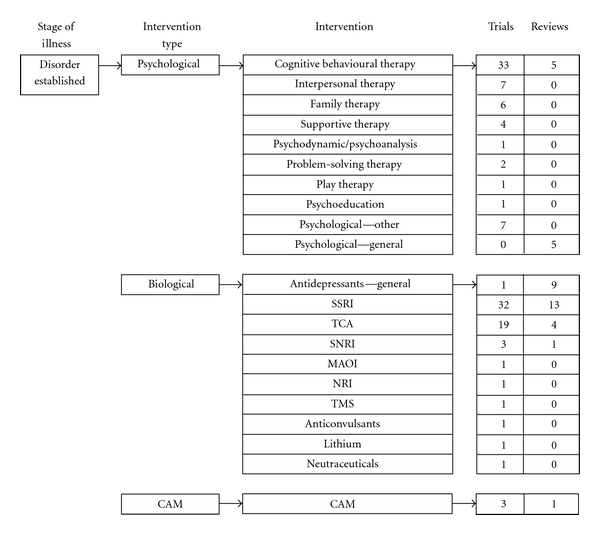
Distribution of included studies to treat a diagnosed depressive disorder.

There were 129 studies of interventions for depressive disorders, of which 81 involved biological interventions (*n* = 56 trials; *n* = 25 systematic reviews), predominantly antidepressant medications (*n* = 78). A total of 58 studies involved psychological interventions (48 trials, 10 systematic reviews) and four studies of complementary and alternative interventions (3 trials, 1 systematic review; Figure 4).

Of the 78 studies investigating medications, the majority involved selective serotonin reuptake inhibitors (SSRIs; *n* = 45). There were fewer trials of tricyclic antidepressants (TCAs; *n* = 23), serotonin-norepinephrine reuptake inhibitors (SNRIs; *n* = 3), and only one trial of monoamine oxidase inhibitors (MAOIs). Of the included systematic reviews, 13 analysed SSRIs, three examined TCAs, and one SNRIs. The earlier reviews assessing TCAs concluded that TCAs were no more effective than placebo in improving depression outcomes [48, 49]. A more recent systematic review reported comparable results, adding that the difference between TCAs and placebo remains nonsignificant with the addition of open-label trials into the meta-analysis [50]. The reviews of SSRIs have highlighted fluoxetine as the SSRI with the most favorable risk-benefit profile [51–54] but there has been vigorous debate about the findings [55, 56]. For example, doubts have been raised about the clinical meaningfulness of the size of the effect in the context of trial participants who are not representative of those typically seen in clinical practice, as well as concerns about methodological aspects of the trials such as high drop-out rates and high rates of placebo response [56]. A meta-analysis including the SNRI venlafaxine concluded that results were inconclusive as trials had not adequately assessed the safety and efficacy of this drug [57].

Of the 58 studies of psychological interventions, CBT was by far the most well studied (33 trials and 5 systematic reviews). There were fewer intervention studies of other psychological therapies in young people, with 7 trials for IPT and 6 for family therapy. There were no published systematic reviews for these treatment modalities alone. One trial examined psychodynamic psychotherapy. The most recent review included in the map [58] was of psychotherapies and 25 of 35 comparisons investigated the effectiveness of CBT, including the more well known of the named CBT programs that is delivered in group format called the “Adolescent Coping with Depression Course” [59–61]. Comparison groups were mostly waiting list control or no treatment groups. Most of the participants were female adolescents with mild to moderate depression recruited from schools. Psychotherapy overall was superior to control conditions after intervention with an NNT of 4.3, as was CBT, behavioural therapy, and interpersonal therapy; however, this evidence of the effectiveness did not remain at longer-term followup [58].

There were 10 trials that compared a psychological therapy with an antidepressant medication and/or a combination of the two, including the very well-known Treatment for Adolescent Depression (TADS) trial. The therapy arms of the 10 trials all had a CBT underpinning, and the medications were all SSRIs, except one that assessed venlafaxine. There were no systematic reviews identified comparing psychological therapies with antidepressant medications and/or a combination of a psychological therapy plus a medication.

### 3.5. Relapse Prevention and Interventions for Persistent Depression

Research into relapse prevention among young people with depressive disorders is limited. Four trials have assessed SSRIs in relapse prevention and one CBT. No systematic reviews were identified. There is a similar paucity of studies investigating interventions for young people with persistent depressive disorders, with only two trials identified, one of which investigated the efficacy of antidepressant medication in the context of a hospital admission [62] and one, the TORDIA study, which assessed the efficacy of combined antidepressants and CBT [63].

## 4. Discussion

Mapping methodology allows for a clear presentation of the nature and extent of high-quality interventions in a broad research field. The evidence map for depression in young people reveals the considerable research into CBT for the prevention of depression, and both CBT and SSRIs for those with a diagnosed depressive disorder, while simultaneously exposing the numerous opportunities for innovative research in this area, most notably for other evidence-based psychological treatments for established disorders (e.g., IPT, problem-solving therapy, and family therapy) and research more broadly into relapse prevention strategies in this population.

### 4.1. Opportunities for Future Prevention Research

There is a considerable literature for prevention research, which is currently dominated by studies of psychological interventions, and in particular those based on CBT. Systematic reviews, while showing the potential to effectively prevent depression in young people, indicate highly variable results from different individual trials. Future research should investigate the most efficacious CBT programs or “active ingredients” of these programs in order to further refine or simplify the interventions.

Future research into the prevention of depression should also consider both new therapeutic approaches and less intensive approaches. Although CBT is a well-founded therapeutic therapy, a narrow focus on this approach could potentially delay significant advancement in the field of preventive interventions. This is particularly the case given that, in many countries, CBT can only be provided by a clinical or registered psychologist. By virtue of the shortage of psychologists in many communities, some new initiatives have focused on delivering CBT by nonpsychologists such as the Improving Access to Psychological Therapies in the United Kingdom [64]. Other alternatives are to assess the efficacy of simpler interventions such as psychoeducation, or problem solving therapy (PST), both of which have been shown to be effective tools in helping to reduce depressive symptoms in young people [65, 66] but currently lack the breadth of high-quality research devoted to CBT. That low-intensity interventions such as psychoeducation and PST have the potential to be delivered by nonmental health specialists, such as teachers or general medical practitioners, will increase the scope for their uptake in the community, which is crucial for prevention strategies [67]. More intensive approaches such as IPT, family therapy, or acceptance and commitment therapy may also be beneficial as potential as preventative interventions, particularly for those young people identified as being at risk of developing depression, although this is yet to be adequately explored in clinical trials.

Finally, given many young peoples' enthusiasm for modern technology, there is potential for more quality research into the efficacy of intervention delivered using electronic technologies, including SMS, email, and Internet-based programs [68, 69]. Delivery of interventions such as psychoeducation, PST, or CBT via electronic means to both populations at risk of depressive disorders, as well as those targeted by universal prevention programs, appears warranted given the prevalence of depressive symptoms.

### 4.2. Opportunities for Research for Those with Established Depressive Illnesses

Intervention studies among young people with diagnosed depression have been dominated by trials of antidepressant medications and CBT trials. Trials and reviews of SSRIs are the most commonly studies biological intervention and generally conclude that there is evidence for the effectiveness of fluoxetine compared with placebo, with inconsistent evidence for other medications in this class [51–54, 70]. However the clinical significance of the results from these trials have been described as far from compelling, with criticisms directed at methodological aspects of the trials including high drop-out rates and high placebo-response rates, concerns about the measurement tools used with questions raised about whether the difference in effect between SSRIs and placebo reflects a difference that is of clinical importance to patients, and concerns about the representativeness of the included participants [54]. A newer class of antidepressants, the SNRIs, have only been subject to three RCTs. While there is considerable research comparing antidepressants to placebo in young people, there is a paucity of research comparing antidepressants head to head, which is an area for greater enquiry, particularly given the relevance to clinicians when deciding which medication to choose.

In terms of psychological interventions, there is again scope to extend the high-quality research beyond CBT to examine other interventions (both simple and more complex), particularly for early or first episodes of disorder, or for less severe depressive disorders in young people. These might include, for example, supportive case management without formal intervention, which was shown in the ADAPT trial to be beneficial for 21% of participants, who were excluded from the intervention phase of the trial because they responded to this brief initial intervention [71]. The National Institute for Health and Clinical Excellence (NICE) guidelines for the identification and treatment of depression in young people also recommends a range of guided self-help approaches that can be useful at early stages of illness [72]. These include exercise, sleep hygiene, anxiety management, and improved nutrition. There are also biological interventions (neutraceuticals) such as fish oil, light therapy, saffron, and folate that have preliminary evidence of effectiveness in adult populations [73], which could be trialed in young people. These interventions are consistent with guideline recommendations of using low-intensity interventions first, before advancing to more complex interventions delivered by mental health specialists.

Furthermore, before further trials of psychotherapeutic interventions are conducted, systematic reviews of intervention studies for IPT and family therapy should be conducted and used to direct further research into these promising approaches. Finally, while short-term psychodynamic psychotherapy and the newer “third wave” CBT therapies like acceptance and commitment therapy (ACT) have has been well studied in adults with depression [74, 75], these are yet to be tested in young people using high-quality controlled trials.

### 4.3. Opportunities for Research in Relapse Prevention and Persistent Depression Strategies

Early onset depression is associated with higher rates of relapse than adult onset [76]. Of adolescents who experience an episode of depression one-third to one-half will go on to experience subsequent episodes in within four to seven years [77, 78] and hence the need to establish effective relapse prevention approaches is critical. The lack of relapse prevention trails in young people with depression is one of the most striking findings of the evidence map, and one of the most disappointing. In order to avert longer-term disability associated with multiple episodes of illness, or persistent depression, research in this area is urgently required. Such studies should be distinct from standard maintenance phases in RCTs and involve rerandomization of treatment responders (which is acknowledged to require significant sample sizes to overcome power issues). While cognitive, and mindfulness-based, cognitive therapy shows promise in adults (e.g., [79, 80]) potential relapse prevention trials could include not just CBT, but other psychosocial interventions such as family therapy, group programs, and vocational interventions, which have been shown to be effective in young people with first episode psychosis [81].

Finally, while research focusing on interventions for “treatment resistant” depression (e.g., failure to respond to more than 3 treatments) in young people is not surprisingly rare given their age, there are research opportunities for further evaluating repetitive Transcranial magnetic stimulation (rTMS) in young people with severe depression that has not previously responded to adequate trials of CBT and/or antidepressant medications. rTMS is said to change brain activity through energy delivered via the magnetic filed created around the brain area it is applied to. It has shown promise as an intervention for treatment resistant depression in adults with a favorable side effect profile [82–84].

### 4.4. Limitations

A limitation of the current study is that there was no evaluation of the effectiveness of the interventions within the trials, restricting us from drawing any conclusions on intervention efficacy. The primary limitation of the current study is the focus on “gold standard” research, which neglects important information available from studies using designs other than the randomized controlled trial and systematic review. Due to resource constraints, trials were also limited to those published after 1980 in English, and quality appraisal of the included studies could not be undertaken. Nonetheless, this evidence map has systematically demonstrated what high-quality evidence does exist for prevention and treatment interventions for young people with depression and has exposed gaps in the research evidence in order to inform researchers, funding bodies, and policy makers as to the opportunities for future research.

## 5. Conclusions

Depressive disorders are currently the leading cause of years of life lived with disability in developed countries and are projected to be second only to cardiovascular disease as the cause of all disability adjusted life years by 2020 [6]. As the onset of depression frequently occurs during adolescence and young adulthood, there is a pressing need to develop effective interventions both to prevent depression in those at risk, and ameliorate symptoms and restore psychosocial functioning in those that experience a depressive episode. Significant gains in this area may be achieved by focusing more on early intervention in this field, particularly the opportunities for responding more comprehensively to “first episode depression” and relapse prevention in younger clients, which has been demonstrated to be highly effective in the clinical management of early psychosis [85–87]. Currently the bulk of expenditure is geared towards acute treatment of diagnosed conditions [88]; however, early intervention would in all likelihood convey significant savings both economically and also in terms of the personal costs associated with a potentially chronic depressive disorder.
